# The Consumption of Animal and Plant Foods in Areas of High Prevalence of Stroke and Colorectal Cancer

**DOI:** 10.3390/nu15040993

**Published:** 2023-02-16

**Authors:** Kellie E. Mayfield, Julie Plasencia, Morgan Ellithorpe, Raeda K. Anderson, Nicole C. Wright

**Affiliations:** 1 Department of Nutrition, Georgia State University, Atlanta, GA 30302, USA; 2 Department of Dietetics and Human Nutrition, University of Kentucky, Lexington, KY 40506, USA; 3 Department of Communication, University of Delaware, Newark, DE 19716, USA; 4 Virginia C. Crawford Research Institute, Shepherd Center and Department of Sociology, Georgia State University, Atlanta, GA 30302, USA; 5 Department of Epidemiology, School of Public Health, University of Alabama at Birmingham, Birmingham, AL 35294, USA

**Keywords:** stroke, colorectal cancer, meat consumption, health outcomes, mediation, racial and ethnic disparities

## Abstract

Diets of red and processed meat have been reported as important risk factors for developing colorectal cancer. Given the racial and ethnic differences in the incidence of colorectal cancer, patterns of food consumption, and areas of residence, particularly in the South, more data is needed on the relationship between residing in a high stroke area, colorectal cancer incidence levels, and red meat and processed meat consumption. We created online surveys to ascertain meat, red meat, and healthy food consumption levels. We used OLS regression to evaluate the association between residence in Stroke Belt states and colorectal cancer incidence quartiles with food consumption. We further used path analysis using structural equation modeling to evaluate if age, sex, race/ethnicity, income, and comorbidity index mediated the association between residence in the eight-state Stroke Belt, colorectal cancer incidence groups, and meat consumption. Our sample included 923 participants, with 167 (18.1%) residing in the Stroke Belt and 13.9% being in the highest colorectal cancer incidence group. The findings show that residing in a Stroke Belt state is predictive of the consumption of overall meat 0.93 more days per week or red meat 0.55 more days per week compared to those not residing in a Stroke Belt state. These data can be used to develop future diet interventions in these high-risk areas to reduce rates of colorectal cancer and other negative health outcomes.

## 1. Introduction

Chronic diseases associated with meat consumption are costly and unequally distributed. Overconsumption of red and processed meat is a well-established risk factor for multiple types of cancers, particularly colorectal cancer [1,2,3,4,5,6,7,8,9,10,11,12,13,14,15], obesity [16], type 2 diabetes, [17] and cardiovascular disease [14]. In the US, colorectal cancer is the second costliest cancer, accounting for 12.6% of all cancer treatment costs [18]. Red and processed meat (e.g., beef, pork, sausages, and hot dogs) are strongly associated with risk of multiple cancers and mortality [8,9,11,12,14]. Studies show that more than 100–120 g of red meat per day increases the risk of colorectal cancer by up to 24%, and more than 25–30 g of processed meat daily increases colorectal cancer risk by up to 49% [1,19,20].

On average, Americans consumed 186 g of processed meat per week and 284 g of unprocessed red meat per week in 2016 [21]. Consumption volumes differ due to numerous factors, particularly race and ethnicity. Studies show that Black and Hispanic Americans consume more meat, especially red and processed meat, than other groups [22,23]. Non-Hispanic Black Americans consumed the lowest level of unprocessed red meat, whereas Hispanics consumed the highest level of unprocessed red meat [21]. Similarly, cancer incidence and mortality rates differ by type of cancer, ethnicity, and race. Hispanic Americans have a greater risk for liver and stomach cancers compared to White Americans [24], and they have equal or lower risk for colorectal [25], breast [26], and ovarian cancers [27]. Hispanic Americans have a higher risk than non-Hispanic White Americans for obesity [28,29,30,31] and diabetes [28,32,33]. However, Black Americans and Hispanic Americans have greater risk than non-Hispanic White Americans for obesity [28,29,30,34,35], diabetes [33,35,36,37], cardiovascular disease [28,35,38,39,40], and both cancer incidence and mortality [41,42].

Certain U.S. regions have higher rates of diet-related diseases. Evidence regarding the concomitant incidence of colorectal cancer and heart disease by region is limited. Stroke is a leading cause of mortality and is of particular concern in what is now labeled the Stroke Belt, which is composed of southern U.S. states. Treatments for stroke is expensive, costing over 50 billion between 2017 and 2018, and it is a contributing factor to long-term disability [43]. The highest concentrations of stroke deaths are mostly in the southeast region, with some of the heaviest concentration in the Deep South, a region of eight states that includes Alabama, Arkansas, Georgia, Louisiana, Mississippi, North Carolina, South Carolina, and Tennessee [44]. Geographic location matters because the south and southeast regions of the U.S. are also where the majority of its Black and Hispanic populations reside [45,46]. Evidence also shows that red meat consumption is a leading risk factor for type 2 diabetes in the U.S. and other parts of the world, such as Australasia [47].

The consumption of healthy foods, such as leafy greens and starches, is associated with reduced stroke risk [48], and evidence regarding the contribution of meat consumption, especially red and processed meat, to risk of stroke is weak [49]. Furthermore, adoption of alternative and plant-based diets such as pescatarian (no meats, except seafood), vegetarian (no meat, but eggs and dairy are included), and vegan (no animal or foods that are sourced from animals) are associated with lower morbidity and mortality from cancer, diabetes, and cardiovascular disease, as well as with reduced diet-related risk factors such as body mass index (BMI), cholesterol, and blood pressure [50,51,52].

Studies show that healthier diet patterns consisting of plant foods can reduce the risk and prevalence of chronic diseases such as cardiovascular disease, hypertension, and some cancers. However, the relationship between living in the Stroke Belt, CRC rates, and diet-related diseases to race/ethnicity is less clear. Therefore, using a nationally representative sample of Hispanics, Blacks, and non-Hispanic Whites, this cross-sectional study examined the association between Stroke Belt residence and colorectal cancer incidence and diet, and it evaluated if age, race/ethnicity, income, and number of health conditions mediates the association between Stroke Belt residence and colorectal cancer incidence with food consumption.

## 2. Materials and Methods

The study was conducted in accordance with the Declaration of Helsinki, and it was determined exempt (category 2) by the Institutional Review Board of Michigan State University. A priori, our intention was to recruit a stratified sample of approximately equal numbers of Black, Hispanic, and non-Hispanic White participants. We first used Amazon’s Mechanical Turk (MTurk) [53], but data has found that the MTurk participant pool tends to overrepresent Whites compared to the general U.S. population [54]; thus, we also employed a second survey tool from Qualtrics Research Suite (Qualtrics, 2009, Provo, UT, USA. Version 12.018). Qualtrics maintains a non-probability, nationally representative panel based on airline lists, online shopping centers, and targeted customer profiles. Participants of this sample are compensated based on their preferred method of compensation (e.g., points redeemable for flyer miles, shopping points, and cash rewards). An e-mail was sent to their panelists who identified themselves as non-Hispanic White, non-Hispanic Black, and Hispanics living in the U.S. For both MTurk and Qualtrics, eligible and interested participants were provided with the link to the informed consent form for participation followed by the web-based survey that included sociodemographic questions. The combined datasets yielded a total of n = 1069. After combining samples, we excluded participants with missing demographic, state, or diet data. The final sample size was 923 total participants; 661 (71.6%) identified as female, the median age was 38.3 years (SD = 15.0, range 18 to 86), 261 (28.3%) were non-Hispanic Black or African American, 278 (30.1%) were Hispanic, and 384 (41.6%) were non-Hispanic White. For additional participant statistics, please see Table 1.

**Colorectal cancer Incidence.** Colorectal cancer incidence data was based on colorectal cancer rates per 100,000 people from 2015 data. We grouped states into quartiles based on colorectal cancer incidence, which included the following: Q4, the highest colorectal cancer incidence (≥42.3), included Alaska, Alabama, Mississippi, Louisiana, Arkansas, West Virginia, Ohio, Kentucky, Illinois, Iowa, North Dakota, and Nebraska; Q3, the second-highest colorectal cancer incidence (41.9–38.7), included Montana, South Dakota, Oklahoma, Missouri, Indiana, Tennessee, Georgia, South Carolina, Pennsylvania, Delaware, New Jersey, and New York; Q2, the second-lowest colorectal cancer incidence (38.4–35.0), included California, Idaho, Texas, Kansas, Minnesota, Wisconsin, Michigan, North Carolina, Maryland, Connecticut, Massachusetts, New Hampshire, and Maine; Q1, the lowest colorectal cancer incidence (<34.9), included Florida, New Mexico, Arizona, Nevada, Oregon, Washington, Wyoming, Utah, Colorado, Virginia, Vermont, and Rhode Island.

**Stroke belt states.** Participants provided their state of residence, and this information was used to delineate whether a participant was living in a Stroke Belt state, which was defined as residing in one of the following eight states: Alabama, Arkansas, Georgia, Louisiana, Mississippi, North Carolina, South Carolina, or Tennessee; n = 167 (18.1%) of the sample resided in a Stroke Belt state.

**Food consumption.** Participants were provided with a list of food categories and were asked to indicate in how many days in the past week they had consumed each food. We grouped foods into the following categories: (1) meat (beef, pork, poultry, fish, and venison), (2) red meat (beef, pork, and venison only), and (3) healthy foods (fruits, leafy green vegetables, starchy vegetables, grains, nuts and seeds, tofu, seitan, and tempeh). We calculated the sum of the days each food group was consumed in the past week. This means that the more foods in each category a person reported consuming over more days in the past week, the higher their score was on the measure. Eggs and dairy were also measured but were not included in these measures due to their controversiality in being included in measures of healthy foods, and they do not belong in either meat category.

**Health condition diagnoses.** Participants were asked to indicate whether they had ever been diagnosed with any of the following health conditions: heart disease, diabetes, high blood pressure, high cholesterol, liver disease, or kidney disease. These conditions were selected as being some that are well-known to be associated with meat consumption. The number of “yes” responses was summed to create an overall measure of health conditions. Those who responded yes to more than three were coded together with those who had three, as only one or two people had scores of four, five, or six; therefore, the final measure was scaled from zero to three as a count of the number of health conditions previously demonstrated to be associated with meat consumption (M = 0.59, SD = 0.87).

**Covariates.** Other variables of interest included age, gender, race/ethnicity, annual household income, and history of any of the following health conditions: heart disease, diabetes, high blood pressure, high cholesterol, liver disease, or kidney disease, which were self-reported by the participants. We counted the number of conditions reported and summed them together to create an overall measure of health conditions ranging from 0 to 3. Sample source (Qualtrics, n = 661, 71.6%, and MTurk, n = 262, 28.4%) was also included as a covariate in the analyses.

**Statistical Analysis.** The characteristics of the sample were compared, and the means and standard deviations (SD) for continuous variables and the frequency and proportion for categorical variables are reported. The sum of the days in which the participants ate each food group in the past week (meat, red meat, and healthy foods) overall, by Stroke Belt residence, and by CRC incidence quartile were estimated, and a *t*-test or ANOVA was used to compare the means within each variable. OLS regressions were used to evaluate the association between Stroke Belt residence and CRC incidence with food consumption. Models for each food group were ran separately. Models were adjusted for age, sex, race/ethnicity, income, and comorbidity score. Subsequently, a path analysis was conducted using structural equation modeling to test mediation effects. Models for overall meat and red meat were ran separately due to the nested nature of the measures. Indirect effects were assessed using 5000 bias-corrected bootstrap samples. We used Stata v.17 (College Station, TX, USA) for all analyses, with the level of significance set to 0.05. The full statistical results can be found in Table 2.

## 3. Results

After excluding 397 participants with missing data, our final sample included 923 participants, of which the mean age was 38.3 years (SD = 15.0, range 18 to 86), 661 (71.6%) were female, 262 (28.4%) were male, 261 (28.3%) were Black, 278 (30.1%) were Hispanic, 384 (41.6%) were non-Hispanic White, 167 (18.1%) resided in the Stroke Belt, 25.2% were in the lowest colorectal cancer incidence group, and 13.9% were in the highest colorectal cancer incidence group (Table 1).

### 3.1. Dietary Habits

Overall, participants consumed 6.95 servings of meat on average in the previous week, with red meat accounting for 3.42 of those servings. About 15.4 servings of healthy foods were consumed in the previous week. As shown in Table 2, Stroke Belt state residence was significantly associated with increased overall meat consumption and red meat consumption but not with healthy food consumption. Colorectal cancer state residence was not significantly associated with meat or red meat consumption, but residence in the second quartile colorectal cancer states was associated with the highest healthy food consumption.

### 3.2. Relationship between Stroke Belt Residence, Colorectal Cancer Incidence, and Food Consumption

Residing in a Stroke Belt state was significantly associated with consuming more meat overall, as well as more red meat, in the past week. There was no significant relationship between Stroke Belt states and healthy food consumption. Colorectal state quartile was not significantly associated with any food consumption outcome, except for living in a second quartile state was associated with higher healthy food consumption, but once covariates were accounted for this relationship was no longer significant. Overall meat consumption and red meat consumption were both significantly associated with increased health conditions reported. The indirect effect of residing in a Stroke Belt state on health conditions was significant when mediated by overall meat consumption (*b* = 0.02, 95% CI (0.002, 0.04)) and by red meat consumption (*b* = 0.01, 95% CI (0.0002, 0.04)) but not by healthy food consumption (*b* = −0.003, 95% CI (−0.02, 0.002)). This suggests that the relationship between residing in a Stroke Belt state and health outcomes may be at least partially mediated by meat consumption. There was not, however, a relationship between CRC quartile and health outcomes.

In the crude analyses, Stroke Belt state residence was associated with more overall meat and red meat consumption. Residing in a Stroke Belt state was significantly associated with consuming more meat overall (*b* = 0.76 (0.38)) and more red meat (*b* = 0.48 (0.25)) in the past week (Table 3). After adjusting for age, sex, income, health conditions, and sample source, we found similar results.

Sample source was included as a covariate after independent-samples *t*-tests found a significant difference between sample sources in the frequency of healthy food consumption (Qualtrics M = 14.44, SD = 7.95; MTurk M = 17.70, SD = 7.03, t (921) = −5.80, *p* < 0.001). There was not a significant difference between sample sources for amounts of meat consumed (Qualtrics M = 6.88, SD = 4.82; MTurk M = 7.11, SD = 4.34, t (921) = −0.65, *p* = 0.51,) or for amount of red meat consumed (Qualtrics M = 3.36, SD = 3.07; MTurk M = 3.56, SD = 2.92, t (921) = −0.91, *p* = 0.36). Of the other covariates, age, gender, race/ethnicity, and income were all significant covariates predicting meat consumption and red meat consumption, but only income was a significant covariate for healthy food consumption. The measures of these covariates are as follows: age on meat, b = −0.04, 95% confidence interval (CI) (−0.06, −0.01); age on red meat, b = −0.03, 95% CI (−0.05, −0.02); age on healthy food, b = 0.01, 95% CI (−0.02, 0.05); gender on meat, b = −1.57, 95% CI (−2.25, −0.88); gender on red meat, b = −1.14, 95% CI (−1.58 −0.70); gender on healthy food, b = −0.01, 95% CI (−1.15, 1.13); Black compared to White on meat, b = 1.54, 95% CI (0.78, 2.30); Black compared to White on red meat, b = 0.55, 95% CI (0.06, 1.04); Black compared to White on healthy food, b = −0.34, 95% CI (−1.62, 0.94); Hispanic compared to White on meat, b = 1.09, 95% CI (0.31, 1.87); Hispanic compared to White on red meat, b = 0.46, 95% CI (−0.05, 0.96); Hispanic compared to White on healthy food, b = 0.45, 95% CI (−0.85, 1.76); income on meat, b = 0.34, 95% CI (0.16, 0.53); income on red meat, b = 0.15, 95% CI (0.03, 0.27); income on healthy food, b = 0.75, 95% CI (0.44, 1.06).

## 4. Discussion

In this cross-sectional study of a representative U.S. sample of non-Hispanic Black Americans, non-Hispanic White Americans, and Hispanic Americans, we found that living in the eight-state Stroke Belt region was associated with eating more meat, especially red meat. The same associations were not seen for colorectal cancer quartiles and meat consumption, nor were there associations between the Stroke Belt region and healthy food consumption. The key findings in this study were that Stroke Belt residency was associated with meat and red meat consumption but not with healthy food consumption, and colorectal cancer state residency was not associated with the diet patterns assessed in this study.

In addition to the findings regarding residence in a Stroke Belt or colorectal cancer state and meat consumption, our results also showed that the health conditions of heart disease, diabetes, high blood pressure, high cholesterol, liver disease, and kidney disease were associated with Stroke Belt residence as mediated by overall meat consumption. These findings are consistent with findings from other, larger studies where a review of epidemiological studies found an association between cardiovascular disease and type 2 diabetes as well as long-term consumption of red and processed meat [55]. Although studies show associations, there is a lack of evidence for the specific components of meat products that increase risks for cardiovascular disease and type 2 diabetes. There is an opening for future research to investigate this finding in more detail in order to better pinpoint the mechanisms and boundary conditions at play in this context.

Compared to White participants, Black participants did report significantly more overall meat and red meat consumption. The results were greater for Black participants compared to the Hispanic participants for both types of meat consumption, though the difference in overall meat consumption was not significant for Hispanics (see Table 2). Other studies showed that when compared to White women, Black women were significantly more likely to eat more total meat [56]. Additionally, compared to White Americans, Black Americans consumed more pork, and Mexican Americans consumed more beef compared to White Americans [23]. These findings were among a youth sample of over 14,000, ages 2 to 18 years, which is much younger than our sample, which had a median age of 38 years [23]. In this study, Hispanic youth did not report significantly more red meat consumption compared to White participants [23]. This is counter to literature reporting that Hispanics eat slightly more red meat than Whites or Blacks [57]. There are many complex factors at play in disparities in meat consumption by racial and ethnic identity. This includes the multifaceted relationships between identity and socioeconomic status, health care access, likelihood of residing in a food- and nutrition-insecure area, lifetime experience of allostatic stress and discrimination, and cultural differences in beliefs and practices surrounding food and nutrition.

A study examining household purchasing patterns, including the effect of spending on red meat on greenhouse gas emissions and diet quality [58] found that greenhouse gas emissions were lower and diet quality was higher in households that spent the least on red meat [58]. Although this study found no differences across race and ethnicity, lower education and study participants enrolled in SNAP were spending a larger percentage of their food income on red meat [58]. Similar to our findings, as income increases, healthier foods are preferred, and thus, a higher diet quality is attained.

When examining cultural factors, traditional or heritage foods may be influential in dietary practices. Within Hispanic ethnic groups, differences in meat consumption exist, and the risk of diet-related diseases differs. For example, in a study conducted in Costa Rica, there was a positive association between unprocessed and processed red meat and abdominal obesity, but this relationship only existed for overall meat consumption and metabolic syndrome [59]. Although this was not a U.S.-based study, immigrants and other ethnic groups build dietary practices around their heritage or traditional foods and are largely dependent on access and affordability. The percent of income spent on food in 2021 in the U.S. was 6.7% versus 31.6% in Costa Rica [60]. These differences may also be seen based on the type of retailers where Americans shop for food. Structural inequities may be more impactful risk factors on diet quality and health due to their impact on the food environment.

Some limitations should be considered. First, the retrospective, self-reported measure of meat consumption has limitations. Research participants often underreport diet information due to reliance on memory of prior intake [61]. The format of the measure was also not ideal, as averaging the number of days that each type of meat was consumed could be interpreted as the variety of meat consumption in addition to the amount or frequency. Although the measure is not ideal as a measure of absolute meat consumption, prior work examining the diet compositions of U.S. ethnic groups supports the self-reported food intake in the current study [23]. Second, we did not specifically ask about processed meat such as hot dogs, sausages, or bacon, nor did we collect information on preparation methods. According to the International Agency for Research on Cancer, red meat consumption and processed meat consumption are probably carcinogenic and carcinogenic to humans, respectively [62]. Although processed meat could not be analyzed in concert with red meat, evidence exists showing that the consumption of red meat is associated with cardiovascular disease mortality [63]. Additionally, a recent study showed that a higher intake of red and processed meat was associated with higher risk of cardiovascular events, but not poultry [63]. Third, dairy foods were not included as part of the analysis of the Stroke Belt and colorectal cancer. Dairy consumption is inversely associated with colorectal cancer and stroke [64,65], and data was not accessed nor was dairy included in our list of healthy foods. Fourth, we did not ask about disability status. A stroke-associated disability may have yielded higher gender differences in the southern Stroke Belt region, which is telling because the percentage of people with disabilities is highest in the southern U.S. [66]. Future studies are needed to address these important issues.

Colorectal cancer and Stroke Belt states are also some of the states with poor access to healthcare, making prevention through not only diet but also regular medical screenings more challenging [67]. Future studies should examine how access to preventative medical care and healthcare professionals confounds poor dietary choices associated with chronic diseases.

## 5. Conclusions 

Health outcomes such as cancer and heart disease have a multitude of public health factors that predict their occurrence. It is known from previous research that meat consumption, especially red and processed meat, is one such factor [55,62]. Geographic location, such as state of residence in the U.S., can also be implicated as a risk factor in many related health outcomes, such as stroke and colorectal cancer incidence [44]. The present study suggests that overall and red meat consumption is associated with geographic location. Public health interventions aimed at reducing diet-related health disparities should consider the confluence of location and meat consumption in the development of lifestyle behavior change strategies and targeting practices. Dietary habits are inextricably linked to systemic and structural influences, which highlights the importance of not only continuing to examine the association of diet choices linked to diet-related health issues, but also to identify protective factors that can be incorporated in public health interventions.

## Figures and Tables

**Table 1:** Participant demographics and characteristics. Note: colorectal cancer is abbreviated in this and all other tables as “CRC”.

	Total n = 923
Sample Source, n (%)	
MTurk	262 (28.4)
Qualtrics	661 (71.6)
Age, mean years (range)	38.3 (18–86)
Female, n (%)	661 (71.61)
Race and ethnicity, n (%)	
Non-Hispanic White	384 (41.6)
Black Americans	261 (28.3)
Hispanic	278 (30.1)
Income, n (%)	
<$25,000	243 (26.3)
$25,000–$50,000	307 (33.3)
$50,001–$75,000	191 (20.7)
$75,001-$100,000	84 (9.1)
>$100,000	98 (10.6)
Residence in Stroke Belt, n (%)	167 (18.1)
Health conditions, mean (SD)	0.49 (0.82)
CRC incidence quartile, n (%)	
1	233 (25.2)
2	359 (38.9)
3	203 (22.0)
4	128 (13.9)

**Table 2:** Dietary habits of participants.

	Overall Meat Mean Days (Range)	*p*-Value	Red Meat Mean Days (Range)	*p*-Value	Healthy Food Mean Days (Range)	*p*-Value
Overall	6.95 (0–35)		3.42 (0–21)		15.4 (0–42)	
Stroke Belt Residence, mean (SD)		0.047		0.050		0.643
Yes	7.60 (5.55)		3.83 (3.61)		15.23 (8.09)	
No	6.84 (4.43)		3.35 (2.85)		15.53 (7.84)	
CRC Incidence Quartile, mean (SD)		0.961		0.708		0.015
1	7.01 (4.24)		3.44 (2.79)		14.93 (8.42)	
2	7.00 (4.67)		3.48 (2.99)		16.36 (7.66)	
3	6.91 (4.99)		3.23 (3.17)		15.01 (7.34)	
4	6.77 (5.12)		3.58 (5.18)		14.09 (8.19)	

**Table 3:** Relationship between Stroke Belt residence, colorectal cancer incidence group, and food consumption.

	Overall Meat		Red Meat		Healthy Food	
	Crude b (95% CI)	Adjusted b (95% CI)	Crude b (95% CI)	Adjusted b (95% CI)	Crude b (95% CI)	Adjusted b (95% CI)
Stroke Belt Residence	0.76 (0.01, 1.52)	1.00 (0.14, 1.86)	0.48 (0.00, 0.97)	0.65 (0.09, 1.21)	−0.30 (−1.59, 0.98)	0.87 (−0.58, 2.31)
CRC Quartile						
1	Ref.	Ref.	Ref.	Ref.	Ref.	Ref.
2	−0.01 (−0.77, 0.75)	0.09 (−0.66, 0.85)	0.04 (−0.045, 0.52)	0.07 (−0.42, 0.56)	1.42 (0.15, 2.69)	1.14 (−0.13, 2.41)
3	−0.10 (−0.97, 0.77)	−0.48 (−1.40, 0.45)	−0.22 (−0.78, 0.34)	−0.41 (−1.00, 0.19)	0.07 (−1.38, 1.52)	−0.12 (−1.67, 1.43)
4	−0.24 (−1.25, 0.76)	−0.24 (−1.28, 0.80)	0.14 (−0.51, 0.78)	0.15 (−0.53, 0.82)	−0.84 (−2.53, 0.84)	−0.74 (−2.49, 1.00)

B: unstandardized coefficient; CI: confidence interval; CRC: colorectal cancer. Adjusted for age, sex, income, health conditions, and sample source.

## Data Availability

Data described in the manuscript will be made available upon request from the corresponding author.
